# Gender Differences in the Perceived Impact of Major Depressive Disorder on Quality of Life: A Cross-Sectional Population Study

**DOI:** 10.3390/jcm14175984

**Published:** 2025-08-24

**Authors:** Cesar Ivan Aviles Gonzalez, Vanessa Barrui, Gian Mario Migliaccio, Felice Curcio, Giovanni Gioiello, Zoraima Romero, Dhurata Ivziku, Sergio Machado, Federica Sancassiani, Diego Primavera

**Affiliations:** 1 Department of Medicine, University of Enna ‘Kore’, 94100 Enna, Italy; 2 Azienda Sanitaria Locale Ogliastra—Sardinia, 08045 Lanusei, Italy; 3 Department of Human Sciences and Promotion of the Quality of Life, San Raffaele Rome Open University, 00166 Rome, Italy; 4 Faculty of Medicine and Surgery, University of Sassari (UNISS), Viale San Pietro 43/B, 07100 Sassari, Italy; 5 PhD Program in Tropical Medicine, Universidad Popular del Cesar, Valledupar 200001, Colombia; 6 Department of Health Professions, Fondazione Policlinico Universitario Campus Bio-Medico, 00128 Rome, Italy; 7 Institute of Psychiatry-IPUB, Federal University of Rio de Janeiro, Rio de Janeiro 22290-140, Brazil; 8 Department of Medical Sciences and Public Health, University of Cagliari, Monserrato Blocco I (CA), 09042 Cagliari, Italy

**Keywords:** major depressive disorder, gender differences, health-related quality of life, SF-12, subjective burden, male depression, mental health, suicide risk, psychiatric comorbidity, help-seeking behaviour

## Abstract

**Introduction:** Major depressive disorder (MDD) is more prevalent in women, but men with MDD may experience higher suicide risk and a different symptom profile. This study investigates the subjective impact of MDD on health-related quality of life (HR-QoL) in males and females. **Methods:** A cross-sectional analysis was conducted on a representative sample from six Italian regions. MDD diagnoses were determined through semi-structured clinical interviews, and HR-QoL was assessed using the SF-12 questionnaire. Mania, hypomania, and subthreshold hypomanic symptoms were evaluated using the Mood Disorder Questionnaire (MDQ). **Results:** Women had a higher prevalence of MDD (6.2%) than men (3.5%). However, men with MDD showed significantly lower HR-QoL scores compared to non-depressed males, with a greater difference than that observed in women. No significant sex differences emerged in psychiatric comorbidities, but men showed a trend toward higher MDQ positivity, possibly indicating a different depressive phenotype. **Conclusions:** Although less frequently diagnosed in men, MDD appears to have a stronger perceived impact on quality of life in males. This finding may reflect under-recognized symptoms such as irritability, hyperactivity, and social rhythm dysregulation. Gender-sensitive screening and intervention strategies are essential to improve early detection and reduce the untreated burden of depression in men, ultimately supporting more equitable mental health outcomes.

## 1. Introduction

Major Depressive Disorder (MDD) is a highly prevalent psychiatric condition that exerts a profound impact on individuals’ psychological functioning, daily life, and overall health. The pathogenesis of depression involves complex interactions between genetic, neurobiological, and environmental factors, including chronic stress, inflammation, and alterations in synaptic plasticity, as demonstrated in both human and rodent studies [1]. These neurobiological mechanisms underscore that depression is not merely a transient mood disturbance, but a systemic disorder with far-reaching effects.

Using objective epidemiological metrics such as Disability-Adjusted Life Years (DALYs), the Global Burden of Disease Study 2019 ranked depression as one of the top contributors to non-fatal health loss worldwide, underscoring its vast social and economic implications [2]. The study evaluated 369 diseases in 204 countries and revealed that MDD remains a leading cause of disability throughout the life course. This finding highlights the need for more effective prevention and intervention strategies, targeting not only pharmacological treatment but also public health measures.

Importantly, this burden is not evenly distributed across populations. Li et al. [3], through further analysis of GBD 2019 data (Institute for Health Metrics and Evaluation (IHME). Global Burden of Disease Study 2019 (GBD 2019) Results; IHME, University of Washington: Seattle, WA, USA, 2020. Available online: https://ghdx.healthdata.org/gbd-2019 (accessed on 11 April 2025)) [2], demonstrated that women bear a higher burden of MDD globally, with increased prevalence, severity, and chronicity. Although the gender gap in disease burden has narrowed slightly over the past three decades, women continue to report higher levels of disability. This persistence indicates that biological, social, and structural factors continue to shape women’s experience of mental illness and require gender-sensitive interventions.

Yet, objective measures such as DALYs may not fully capture the subjective suffering and impact on quality of life associated with MDD. While women report more symptoms and are more frequently diagnosed, men often face undetected and undertreated depression, which can manifest in atypical presentations like irritability or substance abuse rather than sadness or anhedonia. This diagnostic pattern often makes subjective male discomfort invisible, which contributes to the real magnitude of emotional suffering in men not being perceived.

The link between depression and suicide is particularly critical. According to the World Health Organization, more than 60% of global suicides are associated with MDD [4,5], reflecting the disorder’s lethality when untreated. Although women have higher rates of suicide attempts and reported distress, it is men who exhibit significantly higher rates of completed suicides [6]. This is known as the “gender paradox” in suicidality, which reveals a complex dissociation between symptom reporting and suicide completion. While women tend to seek help and use less lethal methods, men use more violent and decisive strategies.

Schrijvers et al. and Kovess-Masfety et al. [7,8] further explain that men often under-report symptoms and delay seeking help due to social stigma and gender norms that equate vulnerability with weakness. This cultural construction of toxic masculinity limits emotional expression and reinforces dysfunctional coping patterns, such as social isolation and self-denial of suffering.

One contributing factor to the heightened risk in men may be the co-occurrence of alcohol and substance use, which is more prevalent among male populations and often acts as a maladaptive coping mechanism. Data from the National Survey on Drug Use and Health (NSDUH) in the U.S. show higher rates of substance misuse among men, particularly those with underlying mental disorders, which exacerbates depressive symptoms and impairs judgment [9]. This concurrent substance use acts as an additional risk factor for both the negative evolution of depressive disorder and the emergence of impulsive suicidal behaviour.

Moreover, the presence of undiagnosed bipolar spectrum disorders in individuals presenting with depressive episodes—more common in men—may lead to ineffective or even harmful treatments, such as antidepressant monotherapy, thereby increasing suicide risk. The BRIDGE Study demonstrated that a significant proportion of patients with MDD symptoms actually meet the criteria for bipolar disorder upon closer evaluation [10]. This overdiagnosis of MDD and underdiagnosis of bipolar disorder in men underscores the importance of a comprehensive diagnostic evaluation that considers the phenotypic variability of emotional distress across genders.

From a sociocultural lens, male suicidal behaviour may be influenced by traditional notions of masculinity, which discourage emotional expression and help-seeking behaviour. Canetto [11] and Cleary [12] argue that masculinity norms push men toward instrumental and sometimes violent expressions of distress, including suicide, while preventing them from accessing mental health services. In recent years, additional concern has emerged around the influence of radical ideological movements—especially online communities—that promote rigid and extreme ideals of male identity. These narratives may reinforce toxic masculinity, body-related insecurity, and rejection of vulnerability, worsen emotional isolation and diminish men’s likelihood to seek psychological support [13].

Therefore, the aim of this study is to explore whether a diagnosis of Major Depressive Disorder (MDD) has a different impact on men and women in terms of self-perceived health-related quality of life (HRQoL). By using a robust, population-based dataset with clinician-administered semi-structured interviews, we seek to overcome the limitations of self-report bias and diagnostic misclassification. The ultimate goal is to identify gender-sensitive pathways and disparities in the psychosocial burden of depression to inform public policy, improve clinical training, and promote mental health equity.

This research will not only highlight gender inequalities in the experience of depression, but can also guide the development of preventive interventions that are more tailored to the different trajectories men and women experience in their psychological distress. Ultimately, this will contribute to the design of more inclusive, culturally competent, and clinically effective mental health public policies.

## 2. Materials and Methods

### 2.1. Design

The study was based on a cross-sectional community survey conducted across six Italian regions, selected to be representative in terms of geographic, cultural, and economic diversity.

### 2.2. Sample

The database was built using interviews with individuals randomly selected after stratification by sex and age groups (18–24; 25–44; 45–64; ≥65) from municipal records in both rural and urban areas. Further details on methodology and recruitment have been published elsewhere [14]. For this survey, we excluded from the analysis all those records (and so individuals) for which the instance of at least one absent answer to the study tools prevented us from reconstructing the personal data required for this study, or even a single item useful for ICD-10 psychiatric diagnosis or for the scoring of the other tools of the study.

### 2.3. Study Instruments

One ad hoc-based toll was used for recording the demographic data of the interviewed people.

ICD-10 psychiatric diagnoses (ICD-10 mood and anxiety disorders including obsessive compulsive disorder and post-traumatic stress disorder; all eating disorders) were carried out by trained medical doctors or a clinical psychologist with multi-year experience in mental health using the semi-structured clinical interview Advanced Neuropsychiatric Tools and Assessment Schedule, a previously validated tool with high reliability with other semi-structured and structured psychiatric diagnostic interviews [15]. While the ANTAS interview was used for diagnosis, its validity compared to standardized diagnostic tools such as the SCID-5 should be further assessed to ensure diagnostic accuracy.

We adopted the previously validated Italian version [16] of the Mood Disorder Questionnaire (MDQ) [17] to identify lifetime episodes of mania, hypomania, and subthreshold hypomanic symptoms—characterized by increased energy or hyperactivity not attributable to a formal diagnosis of mania or hypomania—as these are known to be effectively screened by the MDQ [18].

Perceived Health Related Quality of Life (H-Qol) was assessed by means of the Health Survey Short Form (SF-12), a tool in which a higher score indicates a good perception of one’s own quality of life according to physical and mental quality components, as described previously in the original publication [19], in which it is specified “Permission to use and reproduce the SF-12 is routinely granted…without charge…” [19].

### 2.4. Ethics

The Italian community survey protocol received ethical clearance from the Ethics Committee of the Italian National Institute of Health (Istituto Superiore di Sanità, Rome, Italy), with approval granted on 1 August 2006. This authorization included an assessment of the methodological soundness and reliability of the tools employed, based on the outcomes observed in the research sample. Prior to participation, each respondent provided written informed consent. The study adhered to the ethical principles outlined in the Declaration of Helsinki and received additional approval from the respective ethics committees of all participating regional centres. All individuals gave written consent after being fully informed about the nature and purpose of the study.

### 2.5. Statistical Analysis

Comparisons of numerical data were conducted by 1-way ANOVA for non-repeated measures, and comparisons on nominal variables were conducted through chi-square or Fisher exact tests.

The burden in the worsening of HR-QoL attributable to MDD was calculated as the difference between the HR-QoL (mean score on the SF-12) in males and females in people from the same database without MDD, and the SF-12 mean score of males and females with MDD.

## 3. Results

A total of 2337 individuals were involved in this study; 1005 were male (43.01%) and 1332 female (56.99%); 998 were <45 years old (42.7%); and 1349 were >44 years old (57.3%). Table 1 shows how the lifetime MDD frequency is higher in women (6.38% vs. 1.99%; OR = 3.36, CI 95% 2.05–5.50). People with lifetime MDD show a lower mean age than those who do not have lifetime MDD, but the difference does not reach statistical significance. Males have a higher frequency of MDD under 45 years of age, whereas women show an inverse trend—but even in this case, the differences do not reach statistical significance (Table 1).

Table 2 shows that there are no differences in the mean scores of the SF-12 questionnaire between males and females (33.60 ± 6.52 in males versus 33.49 ± 6.90; *p* = 0.004); however, due to a lower mean score in males than in females in the reference population (i.e., National normative data from the same database men 39.6 ± 6.3 vs. 37.5 ± 5.9 females, *p* < 0.0001), the weight attributable to depression in the worsening of quality of life (mean score in the reference population—mean score in the analysed sample of people with MMD) is greater in males with a statistically significant difference (6.00 ± 5.40 versus 4.01 ± 3.60; one-way 1 GL ANOVA, F = 4.061, *p* = 0.046).

Table 3 inquiries about possible differences in comorbid Mental Health conditions between male and females. Both the mean score of the Mood Disorder Questionnaire (MDQ) and the number of positive responses to the screening tended to be higher among males, but the difference was not statistically significant. The association with eating disorders and, to a lesser extent, with anxiety disorders, tended to be higher in females, but the differences did not reach statistical significance.

## 4. Discussion

The study shows that having a major depressive disorder was associated in a worsening of the perception of quality of life that was stronger in men than in women. Nevertheless, the literature shows that women with MDD often report more intense symptoms compared to men [20], and even that women are more likely to experience recurrent episodes and longer durations of illness with a higher risk of chronicity [21]. These findings suggest that gender differences play a significant role in the manifestation and progression of MDD, but our results suggest that the same is found in the subjective impact of the disorder in the opposite direction, and apparently paradoxically showing a higher subjective impact in men.

The profile of psychiatric comorbidities cannot explain the difference in their subjective impact; in our sample, in fact, there are no significant differences between males and females in the frequency of the comorbidity of anxiety disorders or eating disorders, but rather, a trend towards a higher frequency of these disorders in women with major depressive disorder is evident, although the difference does not reach statistical significance. On the other hand, it is known from larger samples that major depression and mood disorders are associated with anxiety disorders and eating disorders more frequently in women than in men [22,23]. It is therefore reasonable that the differences detected in our sample regarding the comorbidity of anxiety and depression disorders between males and females are the result of a real difference that does not reach statistical significance only due to the small size of the sample and the poor power of the study. However, this consideration is probably also valid for the differences found in the frequency of MDQ positivity in the sexes. In fact, in the Italian general community sample from which this study is drawn, the absolute frequency of MDQ positivity was 3.4% in males versus 2.7% in females [24], and similar results were found in a community sample in Australia in which MDQ-positive males were 61% versus 39% in females [25]. There is, however, only indirect and insufficient scientific evidence to confirm that men with MDD have higher MDQ positivity rates than women with MDD. A study evaluating the Chinese version of the MDQ in patients with mood disorders found that 20.8% were positive. Even this study did not report significant gender differences in MDQ positivity rates among those diagnosed with MDD [26]. However, screening (balanced by sex) did not meet the expectations of a 2–3 to 1 distribution in favour of females (also in those who were given a diagnosis of bipolar disorder 1). Since a clear higher prevalence of women should have been found, it is probably conceivable that positivity to the screening was balanced between the sexes due to a higher frequency of positives among depressed males [26]. In any case, in the absence of robust data, these findings may only produce a heuristic hypothesis of a higher frequency of MDQ positivity in males with depression compared to females. Recent studies have highlighted that the Mood Disorder Questionnaire (MDQ), although having low accuracy as a screener for bipolar disorders due to the high number of false positives [27,28], identifies generic characteristics of hyperactivity and hyper-energy [29,30,31], including people with Dysregulation of Mood, Energy, and Social Rhythms Syndrome (DYMERS) [32,33]. This syndrome is characterized by hyperactivity and dysregulation of social and behavioural rhythms. Additionally, it has been observed that a positive MDQ result is closely associated with sleep rhythm dysregulation, suggesting a link between hyperactivity, rhythm alterations, and irritability symptoms [32,33]. Further research has proposed that DYMERS may represent a common vulnerability to various disorders, including panic disorder, attention deficit hyperactivity disorder, and post-traumatic stress disorder. These disorders share episodes of hyperactivity and dysregulation of social rhythms, even in the absence of a bipolar disorder diagnosis. Such characteristics are often associated with symptoms of irritability [33,34]. Symptoms of irritability “per se” are, however, more frequent in depression in males [10]. Moreover, it has been observed that positivity to the MDQ is associated with a significant deterioration in quality of life, regardless of the presence of psychiatric diagnoses. This suggests that hyperactivity and rhythm dysregulation can have a negative impact on overall well-being, often manifesting with traits of irritability [18]. These studies support the hypothesis that the MDQ may identify individuals with DYMERS, characterized by hyperactivity, rhythm dysregulation, and irritability, even in the absence of a diagnosed bipolar disorder. Irritability and dysphoria together with a greater vulnerability to rhythm dysregulation [31,35], if confirmed to be a characteristic of male depression, could explain a greater impact of depressive disorders in terms of the perception of quality of life in males.

Irritability with a component of hyperactivity and dysphoria, if truly more frequent in male depression, could explain why depression in males, although apparently less serious and with less risk of becoming chronic, can be associated with greater dissatisfaction with quality of life. Dysphoria is in fact often the result of a conflictual relationship with the world and is associated with a lack of self-esteem. The scientific literature provides evidence supporting the association between dysphoria, conflictual relationships, and low self-esteem. Studies have shown that individuals with dysphoria often experience depression and low self-esteem, which can be exacerbated by societal rejection and lack of support [36,37]. Conversely, supportive relationships have been linked to better mental health outcomes in this population [38,39].

In this regard, it is appropriate to distinguish between differences related to biological sex and those attributable to socioeconomic gender, as both may contribute to shaping the subjective impact of depression in men and women. From a biological perspective, there are well-documented differences in brain circuits involved in mood regulation, stress reactivity, and neuroendocrine and inflammatory systems [40,41,42]. In particular, the response of the hypothalamic–pituitary–adrenal (HPA) axis to stressful stimuli and hormonal fluctuations—especially estrogen—appear to contribute to greater vulnerability to depression in women, as well as to increased interpersonal sensitivity and a greater openness to expressing emotional distress [43]. Furthermore, the differential involvement of brain structures such as the amygdala, hippocampus, and prefrontal cortex may modulate the subjective experience of the disorder across sexes [41].

On the other hand, gender differences, understood as cultural and social constructions associated with male and female roles, deeply influence how men and women experience, express, and cope with psychological suffering. In particular, cultural norms that associate masculinity with strength, self-control, and emotional restraint may hinder early identification of distress in men, fostering dysfunctional coping strategies such as social withdrawal, substance use, or denial of suffering [44,45,46,47]. These dynamics may lead to greater internalization of distress and a more pronounced perception of its negative impact on quality of life, even in the absence of severe clinical symptoms.

Moreover, evidence suggests that men are less likely to seek help than women [48], and that hegemonic gender norms may foster atypical forms of male depression that are harder to detect clinically and are associated with higher suicide risk [12,49]. This integrated interpretation—both biological and sociocultural—is consistent with our findings and reinforces the urgency of developing diagnostic and therapeutic approaches that are gender-sensitive.

The role of the more frequent dysphoria with hyperactivity in male depression together with the low perceived quality of life could represent a contributory cause of two relevant factors on a clinical and prevention level; the first is the fact that it is known that males with depression require less help than women [48], the second is the higher frequency of suicide in depressed males [26,50].

## 5. Conclusions

This study provides robust evidence that Major Depressive Disorder (MDD) exerts a greater subjective impact on health-related quality of life (HR-QoL) in men than in women, despite the well-established higher prevalence and chronicity of the disorder in females. While women tend to report more severe and recurrent symptoms, our findings reveal that men with MDD perceive a stronger deterioration in their overall well-being, as measured by the SF-12 questionnaire. This result, although seemingly paradoxical, highlights a gender-based dissociation between diagnostic frequency and lived burden, which deserves closer examination.

Importantly, this disparity cannot be fully explained by the profile of psychiatric comorbidities, as no significant sex-based differences emerged in our sample regarding anxiety or eating disorders. However, men with depression showed a trend toward higher MDQ positivity, possibly reflecting greater rates of dysphoria, irritability, and rhythm dysregulation—dimensions that are increasingly recognized as characteristic of a male-specific depressive phenotype. These traits may correspond to a broader vulnerability profile, such as that proposed in the construct of Dysregulation of Mood, Energy, and Social Rhythms Syndrome (DYMERS), which has been linked to reduced quality of life and may manifest independently of bipolar diagnoses.

Furthermore, this study reinforces the notion that male depression is not only underdiagnosed but also misunderstood, in part due to gendered cultural scripts. Traditional norms of masculinity—and, more recently, radical ideological movements particularly active in online spaces—promote unrealistic and rigid male identity models that suppress emotional expression and stigmatize help-seeking behaviours. These cultural constructions may intensify emotional isolation, contribute to body-related insecurity, and delay access to treatment, ultimately exacerbating the subjective burden of depression in men.

Taken together, our results support the need for gender-sensitive clinical tools and public health strategies. Recognizing that male depression may manifest through symptoms such as hyperactivity, irritability, and emotional detachment—rather than the more “classic” symptoms of sadness or crying—is critical for improving diagnostic accuracy and timely intervention. Moreover, the lower help-seeking behaviour observed in men, combined with their disproportionately higher suicide rates, underscores the urgency of adapting mental health services to be more inclusive and responsive to male-specific needs.

Future research should validate these findings in larger, multi-ethnic samples and explore the interplay between biological sex differences and sociocultural gender constructs. Investigating the contribution of factors such as neuroendocrine regulation, sleep and social rhythm disturbances, and internalized gender norms will be essential to better understand how men experience and report depression. A transdisciplinary approach, integrating neuroscience, psychology, and gender studies, may offer new pathways toward more effective, equitable, and personalized mental health care.

In conclusion, by shedding light on the disproportionate subjective impact of MDD in men, our study challenges conventional paradigms and contributes to a growing body of literature that calls for rethinking the way we conceptualize, diagnose, and treat depression through the lens of gender. Addressing these gaps is not only a matter of clinical relevance but also of public health and social justice.

## Figures and Tables

**Table 1:** Sex- and age-stratified prevalence of MDD and comparison of individuals with and without MDD.

	With MDD	Without MDD	Total	χ^2^/ANOVA 1103 df	*p*	OR (CI 95%)
**Males**	20 (1.99%)	985	1005	25.742	<0.001	Females 3.36 (2.05–5.50)
**Female**	85 (6.38%)	1247	1332			
**Total**	105 (4.49%)	2232	2337			
**Age years**	47.77 ± 16.78	51.21 ± 8.98	48.42 ± 17.08	F = 0.551	0.460	
**<45 males**	8 (1.57%)	502	510	1.165	0.280	>44 M 1.63 (0.66–4.04)
**>44 males**	12 (2.61)	460	495			
**<45 female**	35 (7.17%)	476	488	0.542	0.462	<45 F 1.18 (0.75–1.84)
**>44 female**	50 (6.21%)	804	854			

**Table 2:** Comparison by sex of H-QoL impairment and perceived burden among individuals with lifetime MDD.

	Male	Female	Male vs. Female ANOVA 1103 df	*p*
**SF-12**	with MDD N = 20 33.60 ± 6.52	with MDD N = 85 33.49 ± 6.90	F = 0.004	*p* = 0.940
**National normative mean score esteemed from the same database** **(Carta et al., 2012) [14]**	39.6 ± 6.3	37.5 ± 5.9	ANOVA * F = 99.2	*p* < 0.0001
**Burden due to MDD**	6.00 ± 5.40	4.01 ± 3.60	F = 4.061	*p* = 0.046

* Detailed statistics in Carta et al., 2012 [14].

**Table 3:** Comparison of psychiatric comorbidity frequency by sex in individuals with lifetime MDD.

**MDQ**	1.71 ± 2.60	1.55 ± 2.91	1.67 ± 2.64	ANOVA 1 way, 103 df F = 0.055	0.814
**MDQ+**	21 (6.66%)	6 (7.06%)		Fisher	0.257
**Eating Disorders**	0	4 (4.7%)	4 (3.80%)	Fisher	0.582
**People with Anx DSM_IV**	5 (25%)	29 (34.12%)	34 (32.38%)	χ^2^ 1 df = 0.615	0.433
**With anxiety or eating comorbidity**	5 (25%)	32 (37.6%)	37 (35.24%)	χ^2^ 1 df = 1.797	1.180

## Data Availability

The data presented in this study are available upon request from the corresponding author. Due to privacy and ethical issues, data are not publicly available.
